# Vagus Nerve Stimulation Improves Mitochondrial Dysfunction in Post–cardiac Arrest Syndrome in the Asphyxial Cardiac Arrest Model in Rats

**DOI:** 10.3389/fnins.2022.762007

**Published:** 2022-05-26

**Authors:** Seonghye Kim, Inwon Park, Jae Hyuk Lee, Serin Kim, Dong-Hyun Jang, You Hwan Jo

**Affiliations:** ^1^Department of Emergency Medicine, Seoul National University Bundang Hospital, Seongnam-si, South Korea; ^2^Department of Emergency Medicine, Seoul National University College of Medicine, Seoul, South Korea

**Keywords:** heart arrest, post-cardiac arrest syndrome, vagus nerve stimulation, mitochondria, cell respiration, reperfusion injury

## Abstract

Cerebral mitochondrial dysfunction during post–cardiac arrest syndrome (PCAS) remains unclear, resulting in a lack of therapeutic options that protect against cerebral ischemia–reperfusion injury. We aimed to assess mitochondrial dysfunction in the hippocampus after cardiac arrest and whether vagus nerve stimulation (VNS) can improve mitochondrial dysfunction and neurological outcomes. In an asphyxial cardiac arrest model, male Sprague–Dawley rats were assigned to the vagus nerve isolation (CA) or VNS (CA + VNS) group. Cardiopulmonary resuscitation was performed 450 s after pulseless electrical activity. After the return of spontaneous circulation (ROSC), left cervical VNS was performed for 3 h in the CA + VNS group. Mitochondrial respiratory function was evaluated using high-resolution respirometry of the hippocampal tissue. The neurologic deficit score (NDS) and overall performance category (OPC) were assessed at 24, 48, and 72 h after resuscitation. The leak respiration and oxidative phosphorylation capacity of complex I (OXPHOS CI) at 6 h after ROSC were significantly higher in the CA + VNS group than in the CA group (*p* = 0.0308 and 0.0401, respectively). Compared with the trends of NDS and OPC in the CA group, the trends of those in the CA + VNS group were significantly different, thus suggesting a favorable neurological outcome in the CA + VNS group (*p* = 0.0087 and 0.0064 between times × groups interaction, respectively). VNS ameliorated mitochondrial dysfunction after ROSC and improved neurological outcomes in an asphyxial cardiac arrest rat model.

## Introduction

Restoration of cerebral function is the key factor in the successful resuscitation of patients with out-of-hospital cardiac arrest (Laver et al., 2004; Nolan et al., 2008). Although return of spontaneous circulation (ROSC) is achieved in a proportion of patients with cardiac arrest, more than 90% of these patients experience severe neurological injury (Grasner et al., 2016; Kim et al., 2018; Myat et al., 2018). Several mechanisms have been suggested to explain post–resuscitation cerebral injury and the therapeutic targets (Stub et al., 2011; Sekhon et al., 2017). However, a majority of therapies that target the amelioration of cerebral injury in post–cardiac arrest syndrome (PCAS) have proven unsuccessful, and targeted temperature management remains the only effective treatment (Nolan et al., 2008; Choudhary et al., 2021; Perkins et al., 2021; Sandroni et al., 2021).

Resuscitation results in reperfusion of ischemic tissues, recovery of aerobic metabolism, and organ perfusion; consequently, ischemia–reperfusion injury is inevitable (Madathil et al., 2016). Although mitochondria are known to be key determinants of ischemia–reperfusion injury, cerebral mitochondrial dysfunction during the post–resuscitation period following cardiac arrest is not well understood (Wiberg et al., 2020). Furthermore, most of our understanding of mitochondrial function and energy metabolism is derived from the myocardium (Yeh et al., 2009; Fang et al., 2012) and focal cerebral ischemia models (Li et al., 2012; Madathil et al., 2016). A previous preclinical study in the swine cardiac arrest model reported decreased mitochondrial respiratory coupling and calcium retention capacity with increased reactive oxygen species (ROS) production in the brain (Matsuura et al., 2017). The study also suggested that the current approach of cardiopulmonary resuscitation (CPR) has limited effect on the restoration of the mitochondrial function in the brain; therefore, there is a demand for urgent and novel therapeutic strategies for the restoration of mitochondrial function (Choudhary et al., 2021).

Vagus nerve stimulation (VNS) has been used to treat refractory partial epileptic seizures and treatment-resistant depression (Gonzalez et al., 2019; Lv et al., 2019). VNS has also been proposed to exert anti-inflammatory effects not only in clinical studies but also in preclinical studies on sepsis and stroke (Borovikova et al., 2000; Ay et al., 2016). In a previous study in a rat model of asphyxial cardiac arrest, VNS accelerated the recovery of cerebral blood flow during the post–cardiac arrest period and improved ischemic hippocampal neuronal damage and functional neurological outcomes (Sun et al., 2018; Kim et al., 2019). Furthermore, VNS has been suggested to exert protective effects against mitochondrial dysfunction in the myocardium (Samniang et al., 2016). However, the effects of VNS on cerebral mitochondrial function, particularly during cardiac arrest, have not been fully evaluated.

Therefore, the aim of this study was to investigate the effects of VNS on mitochondrial respiration in an asphyxial cardiac arrest rat model. We hypothesized that VNS could mitigate cerebral mitochondrial dysfunction and exert therapeutic effects to protect against post–resuscitation cerebral injury.

## Materials and Methods

This study was approved by the Institutional Animal Care and Use Committee of the Seoul National University Bundang Hospital (protocol No. BA 1803-243/022-01).

### Experimental Design

**FIGURE 1:**
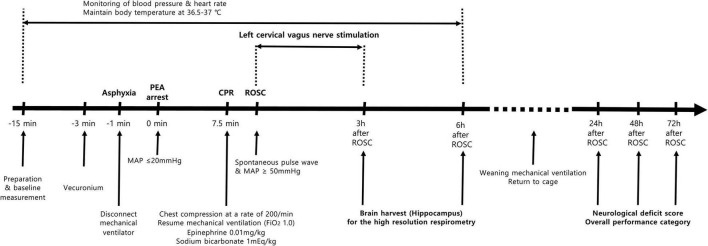
Schematics of the experimental protocol.

A schematic of the experimental protocol is presented in Figure 1. The experiments required two sets of brain harvests for high-resolution respirometry and an evaluation of neurological outcomes.

### Animal Preparation

Male 9-week-old Sprague–Dawley rats weighing 290–320 g were used in this study. The animals were housed in a controlled environment with unrestricted access to food and water before the experiments. They were anesthetized with an intramuscular injection of tiletamine/zolazepam (30 mg/kg; Zoletil, Virbac, France) and xylazine (10 mg/kg, Rompun, Bayer, Germany) and intubated with a 16-gauge catheter (Becton, Dickinson and Company, Franklin Lakes, NJ, United States), which was connected to a ventilator (Harvard rodent ventilator Model 645, Harvard Apparatus, Holliston, MA, United States). The ventilator was set to 2.0 mL of tidal volume with a respiratory rate of 45 breaths per minute. The minute ventilation was adjusted to achieve partial pressure of CO_2_ (PaCO_2_) of 35–40 mmHg on arterial blood gas analysis (ABGA). Body temperature was monitored with a rectal probe and was maintained at 36.5^°^C–37.5^°^C using an infrared heater. Under aseptic conditions, a 24-gauge catheter (Introcan, B. Braun, Germany) was surgically introduced into the left femoral artery on the left inguinal site to monitor the blood pressure and obtain blood samples for ABGA. Resuscitative drugs were administered *via* the left femoral arterial catheter. ABGA was performed before inducing asphyxial cardiac arrest and after weaning from the mechanical ventilator.

### Cardiac Arrest and Resuscitation

To induce asphyxial cardiac arrest, vecuronium (0.2 mg/kg) was injected to paralyze respiration. After the discontinuation of mechanical ventilation, the heart rate (HR) and mean arterial pressure (MAP) increased initially, followed by progressive bradycardia and hypotension. Circulatory arrest was defined by the onset of MAP under 20 mmHg and maintained for 450 s. Following our previously established model (Lee et al., 2013; Kim et al., 2019), cardiac arrest was achieved in less than 120 s of asphyxia. Therefore, if the induction time of cardiac arrest was longer than 120 s, animals were excluded to control the duration of hypoxia. CPR was performed after 450 s of circulatory arrest. It included restarting mechanical ventilation (tidal volume, 2.5 mL; fraction of inspired oxygen (FiO_2)_, 1.0; respiratory rate, 55/min), administering epinephrine (0.01 mg/kg) and bicarbonate (1.0 mEq/kg), and continuous external chest compressions at a rate of 200 compressions per minute using a mechanical thumper (custom-made device, compressed air-driven, rate 200 cycles/min) until spontaneous pulse was detected on the arterial blood pressure monitor and the mean arterial blood pressure exceeded 50 mmHg. Immediately after the ROSC, the animals were randomized into two groups: the vagus nerve isolation group (CA, *n* = 28) and the VNS group (CA + VNS, *n* = 26).

In the post-resuscitative period, body temperature usually decreased with injection of drugs during the resuscitation period was maintained at 36.5^°^C–37.5^°^C using an infrared heater to the extent possible. In the experimental set of brain harvest, additional intramuscular anesthesia was provided to maintain until predefined time of harvest. In the experimental set of neurological outcome assessment, additional anesthesia was not provided and weaning from ventilator was performed after spontaneous respiration was recovered. Adequate spontaneous ventilation, defined as no decrease of mean arterial pressure for 5 min with PaO_2_ > 60 mmHg on room air, was assessed after disconnection of mechanical ventilator. Extubation of the endotracheal tube was then performed and the rats were returned to the cages for neurological observation for 72 h. During the neurological observation period, fluid (5% Dextrose in saline, 50 mL/kg) was subcutaneously administered every 24 h for nutritional support and analgesia (Ketoprofen, 5 mg/kg) was subcutaneously provided if signs of pain were observed.

### Vagus Nerve Stimulation

To stimulate the vagus nerve, the left cervical vagus nerve (VN) was isolated. A 1-cm incision was made 0.5 cm to the left of the midline of the neck. After retracting the muscles, the left carotid artery was exposed, and the left VN was meticulously dissected from the carotid sheath. VN was stimulated using 1-mA pulses of 10-ms duration at 1 Hz for 3 h after ROSC (Model 2100 isolated pulse stimulator, A-M Systems, Sequim, WA, United States) (Kim et al., 2019). Nerve stimulation was performed with a platinum electrode (Plexiglas-platinum electrode, 73-0336, Harvard Apparatus, Holliston, MA, United States), which offers the advantage of stable electrical delivery and protection from the surrounding fluid. A wet gauze of an appropriate level without water leakage was applied to the VNS isolation site to maintain a moist environment, and the wetness level was repeatedly estimated to determine when the gauze should be replaced. In the vagus nerve isolation group (CA), VNS isolation and placement of electrode without stimulation was performed.

### Tissue Preparation

At 3 h (CA_3 h, *n* = 8; CA + VNS_3 h, *n* = 8) and 6 h (CA_6 h, *n* = 8; CA + VNS_6 h, *n* = 7) after ROSC, additional intramuscular anesthesia was provided, and euthanasia was achieved with exsanguination using a femoral arterial catheter in 1 min to prevent tissue swelling. After confirmation of death, a thoracotomy and clamping of the descending aorta were conducted. Then, perfusion with DPBS at 4^°^C using a 20-gauge needle through the left ventricle was performed, and the brain was harvested. The sham group (Sham, *n* = 9) was used to identify the baseline variables of mitochondrial respiration. In the sham group, intubation and femoral artery catheterization were performed, and before asphyxia modeling, euthanasia as described above was achieved for brain harvest. The hippocampus was carefully isolated from the harvested brain tissue and suspended in buffer X with an ice pack. The right hippocampal tissue was dissected with a microblade, and 10 mg of tissue was transferred into 200 μL of assay buffer (50 mL of buffer Z + 0.1 M EGTA + 0.313 g creatine) and homogenized (Perry et al., 2011).

### Measurements of Mitochondrial Respiration

To measure mitochondrial oxygen consumption, high-resolution respirometry was performed (Supplementary Figure 1) (O2k, OROBOROS INSTRUMENT, Innsbruck, Austria) (Gnaiger et al., 2000; Burtscher et al., 2015). The resulting homogenates of hippocampal tissue were diluted and transferred into chambers of calibrated Oxygraph-2K. Oxygen polarography was performed under controlled temperature (37 ± 0.001^°^C) using electronic Peltier regulation. Oxygen concentration (CO_2_, μM) and oxygen flux per tissue mass (pmol O_2_/s*mg) were measured in real time using specialized software (DatLab, OROBOROS INSTRUMENT). According to the current substrate-uncoupler-inhibitor titration (SUIT) protocol, non-phosphorylating leak respiration was induced by adding the CI-linked substrate glutamate (5 mM, G5889, Sigma-Aldrich, St. Louis, MO, United States), malate (2 mM, G7397, Sigma-Aldrich), and pyruvate (5 mM, P2256, Sigma-Aldrich). The oxidative phosphorylation capacity of complex I (OXPHOS C I) activity was measured after adding a saturating concentration of adenosine 5’-diphosphate (ADP) (5 mM, ADP sodium salt, Sigma-Aldrich A2754). OXPHOS capacity combined with CI and II (OXPHOS CI + II) was estimated by adding succinate (10 mM, sodium succinate dibasic hexahydrate, Sigma-Aldrich S2378). Carbonyl cyanide 4-(trifluoromethoxy) phenylhydrazone (FCCP) (0.5 mM, Sigma-Aldrich C2920) triggers proton leakage over the inner mitochondrial membrane. The capacity of the electron transfer system (ETS), which is the non-coupled state at the optimum uncoupler concentration of the maximum oxygen consumption, was measured. Inhibition of CI by rotenone (1 μM, Rotenone, Sigma-Aldrich R8875) was used to measure OXPHOS CII-linked ETS capacity. To control for other oxygen-consuming processes, ETS was inhibited using malonate (5 mM, malonic acid, Sigma-Aldrich M1296) and antimycin (1 μM/mL, antimycin A from Streptomyces sp., Sigma-Aldrich A8674). The consequent residual oxygen consumption (ROX) represents oxygen consumption from undefined sources and was subtracted from the mitochondrial respiratory states.

### Assessment of Neurological Outcomes

Neurological outcomes were evaluated in the CA (*n* = 12) and CA + VNS groups (*n* = 11) at 24, 48, and 72 h after ROSC using the neurological deficit scale (NDS) (0–80; normal = 80; brain death = 0) and overall performance category (OPC) (1 = normal; 2 = slight disability; 3 = severe disability; 4 = comatose; 5 = dead) (Jia et al., 2008; Lee et al., 2013). The scores were measured independently by two researchers blinded to the allocation of the experimental groups, and any discrepancy between their measurements was reviewed by the authors.

### Statistical Analysis

Normality tests were performed using the Kolmogorov–Smirnov test. Variables are presented as mean ± standard deviation (S.D.) or median (Interquartile range), as appropriate. Analysis of variance (ANOVA) with Bonferroni’s *post hoc* multiple comparisons were utilized to compare the variables in the respirometry at each defined period (3 h and 6 h) with the variables in sham. Two-way repeated-measures ANOVA (RM ANOVA) with Bonferroni’s *post hoc* multiple comparisons were used to compare the trends and variables in repeated measurement of neurological outcomes. Due to missing variables, mixed-effects model with Bonferroni’s *post hoc* multiple comparisons were utilized to compare trends and variables in repeated measurements of hemodynamic variables. Statistical significance was set at *p* < 0.05. Statistical analyses were performed using Prism 9.0 (GraphPad Software Inc., San Diego, CA, United States).

## Results

### Baseline Characteristics of the Animals

The baseline characteristics of the two groups (CA vs. CA + VNS) demonstrated no significant differences in body weight, pre-ABGA and post-ABGA values, serum lactate levels, or total ischemia time between the induction of cardiac arrest and ROSC (Table 1). Trends in the mean arterial pressure over the observation period were not significantly different between the two groups (Supplementary Figure 2A; *p* < 0.0001 between times, *p* = 0.0701 between groups, *p* = 0.9914 between times × groups; mixed-effects model). Trends in heart rate over the observation period were significantly different between the two groups (Supplementary Figure 2B; *p* < 0.0001 between times, *p* = 0.6053 between groups, *p* = 0.0234 between times × groups; mixed-effects model). From 90 to 150 min after ROSC, the heart rate tended to decrease in the CA + VNS group compared with that in the CA group, which coincides with the VNS period (Supplementary Figures 2B–D); however, the difference in heart rate during the VNS period was not statistically significant (Supplementary Table 1).

**TABLE 1:** Comparison of hemodynamic and blood gas analysis variables.

Variables	CA (*n* = 28)	CA + VNS (*n* = 26)	*p*-value
Body weight	294.0 [285.0–314.0]	304.0 [295.0–313.0]	0.43
**Hemodynamic and blood gas values at baseline**			
pH	7.38 [7.35–7.40]	7.38 [7.34–7.40]	0.81
PaCO_2_ (mmHg)	36.5 [34.5–44.4]	39.5 [35.8–42.2]	0.62
PaO_2_ (mmHg)	68.1 [58.4–75.2]	73.7 [59.9–80.1]	0.32
HCO_3_^–^ (mmol/L)	22.0 [20.6–24.9]	22.6 [21.3–24.0]	0.87
Base excess (mmol/L)	–3.0 [–5.5–0.1]	–2.4 [–4.1—1.6]	0.95
Lactate (mmol/L)	0.9 [0.8–1.0]	0.9 [0.7–1.1]	0.94
MAP (mmHg)	109.0 [90.0–140.0]	110.0 [78.0–128.0]	0.48
Heart rate (bpm)	287.0 [258.0–313.5]	293.0 [271.3–312.8]	0.48
Body temperature (^°^C)	37.1 [36.9–37.4]	36.6 [36.3–37.2]	0.07
**Hemodynamic and blood gas values at ROSC period**			
ph	7.30 [7.22–7.33]	7.31 [7.22–7.42]	0.25
PaCO_2_ (mmHg)	74.6 [70.1–82.0]	69.8 [60.1–79.1]	0.22
PaO_2_ (mmHg)	115.9 [92.4–157.9]	128.7 [107.7–156.3]	0.25
HCO_3_^–^ (mmol/L)	35.8 [31.4–40.6]	37.1 [32.6–42.2]	0.83
Base excess (mmol/L)	10.1 [4.8–14.0]	11.4 [5.0–16.2]	0.62
Lactate (mmol/L)	7.4 [5.5–7.9]	6.6 [5.9–7.9]	0.78
MAP (mmHg)	112.0 [100.0–131.0]	101.0 [89.8–119.0]	0.08
Heart rate (bpm)	136.0 [116.0–261.0]	128.0 [96.0–165.0]	0.13
Body temperature (^°^C)	34.5 [34.2–34.9]	33.6 [33.1–34.9]	0.07
**Post ROSC 1 h period**			
Body temperature (^°^C)	36.7 [36.4–37.0]	36.3 [35.9–36.9]	0.13
Induction time (s)	73.0 [64.0–79.0]	74.0 [68.0–82.0]	0.41
CPR duration (s)	25.0 [24.0–29.0]	24.0 [22.0–27.0]	0.21
Total ischemia time (s)	550.0 [538.0–556.0]	552.0 [544.0–556.0]	0.21

*Variables are presented with median [IQR]. ROSC, return of spontaneous circulation; MAP, mean arterial pressure.*

### High-Resolution Respirometry

**FIGURE 2:**
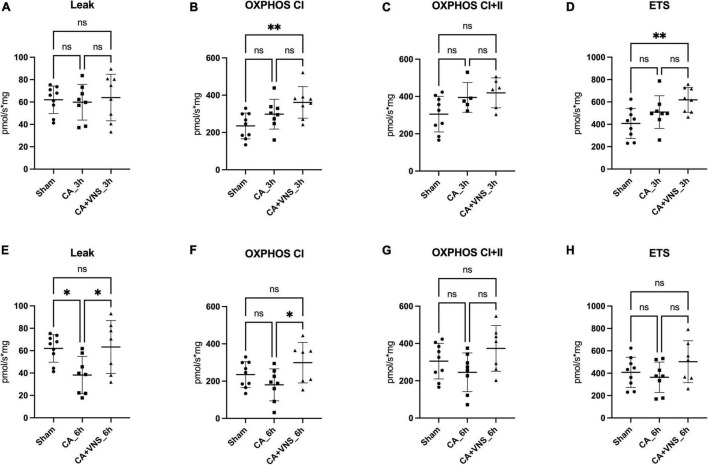
Comparisons of mitochondrial respiration at 3 h and 6 h after ROSC. **(A)** Leak, **(B)** OXPHOS CI, **(C)** OXHPOS CI + II, and **(D)** ETS in the sham group (*n* = 9), CA_3 h group (*n* = 8), and CA + VNS_3 h group (*n* = 8). **(E)** Leak, **(F)** OXPHOS CI, **(G)** OXHPOS CI + II, and **(H)** ETS in the sham group, CA_6 h group (*n* = 8), and CA + VNS_6 h group (*n* = 7). Data are presented as the mean ± standard deviation. One-way ANOVA with Bonferroni’s post hoc multiple comparisons test was performed (*P < 0.05; **P < 0.01; ns, not significant); CA, cardiac arrest; ETS, electron transfer system; OXPHOS, oxidative phosphorylation capacity; VNS, vagal nerve stimulation.

Three hours after ROSC, no significant differences in leak respiration were observed between the sham, CA, and CA + VNS groups (Figure 2A). Complex I respiration (OXPHOS CI), Complex I + II respiration (OXPHOS CI + II), and ETS tended to increase at 3 h after ROSC; however, the difference was not statistically significant (Figures 2B–D). OXPHOS CI and ETS in the CA + VNS group were significantly higher than those in the sham group (*p* = 0.0080 and *p* = 0.0089, respectively) (Figures 2B,D).

Six hours after ROSC, leak respiration was significantly lower in the CA group than in the sham model (Figure 2E) (Sham vs. CA_6 h, *p* = 0.0291) and was restored in the CA + VNS group, which was significantly different from that in the CA group (CA_6 h vs. CA + VNS_6 h, *p* = 0.0308). Complex I respiration (OXPHOS CI) tended to decrease at 6 h after ROSC, while a significant increase was identified in the CA + VNS group (Figure 2F, CA_6 h vs. CA + VNS_6 h, *p* = 0.0401). Complex I + II respiration and ETS capacity tended to decrease at 6 h after ROSC and slightly increased in the CA + VNS group at 6 h after ROSC; however, there was no statistically significant difference (Figures 2G,H).

### Neurological Outcomes

**FIGURE 3:**
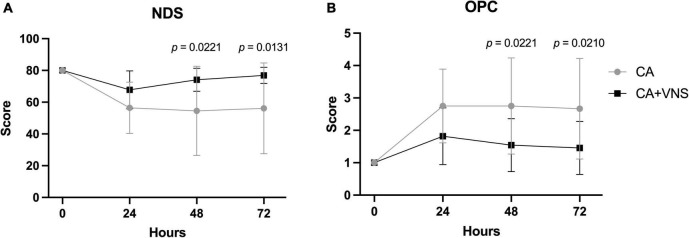
Comparisons of neurological outcomes at 0, 24, 48, and 72 h after ROSC. **(A)** NDS in the CA (*n* = 12) and CA + VNS (*n* = 11) groups at 0, 24, 48, and 72 h after ROSC. *p* < 0.0001 between times, *p* = 0.0300 between groups, *p* = 0.0087 between times × groups; two-way RM ANOVA. **(B)** OPC (OPC; 1, normal; 2, slight disability; 3, severe disability; 4, comatose; 5, dead) in the CA (*n* = 12) and CA + VNS (*n* = 11) groups at 0, 24, 48, and 72 h after ROSC. *p* < 0.0001 between times, *p* = 0.0271 between groups, *p* = 0.0064 between times × groups; two-way RM ANOVA. Data are presented as the mean ± standard deviation. Only *p*-values < 0.05 are depicted in the graph and indicate the statistical comparisons of both groups at the marked time (Bonferroni’s *post hoc* multiple comparisons test). NDS, neurologic deficit score; OPC, overall performance category; ROSC, return of spontaneous circulation; VNS, vagal nerve stimulation; CA, cardiac arrest.

The trends in NDS after ROSC were significantly different between the CA and CA + VNS groups (Figure 3A; *p* < 0.0001 between times, *p* = 0.0300 between groups, *p* = 0.0087 between times × groups; two-way RM ANOVA). NDS at 48 h and 72 h after ROSC were significantly higher in the CA + VNS group than in the CA group (*p* = 0.0221 and *p* = 0.0131, respectively; Bonferroni’s *post hoc* multiple comparisons test). Similarly, the trends in OPC after ROSC were significantly different between the CA and CA + VNS groups (Figure 3B; *p* < 0.0001 between times, *p* = 0.0271 between groups, *p* = 0.0064 between times × groups; two-way RM ANOVA). OPC at 48 h and 72 h after ROSC were significantly lower in the CA + VNS group than in the CA group (*p* = 0.0221 and *p* = 0.0210, respectively; Bonferroni’s *post hoc* multiple comparisons test), thus suggesting favorable neurological outcomes in the CA + VNS group.

## Discussion

In this study, using the asphyxial cardiac arrest model of rats, we identified that VNS might reduce cerebral injury by improving the mitochondrial dysfunction induced by ischemia–reperfusion injury. At 6 h after ROSC, leak respiration was significantly decreased, and CI, CI + II, and ETS were tended to decrease without statistical significance, which were all reversed by VNS. Although the increases in CI + II and ETS were not statistically significant, leak and CI respiration in the CA + VNS group significantly increased at 6 h after ROSC compared to the CA group. Additionally, VNS improved the functional neurological outcomes at 48 and 72 h after ROSC, as measured using NDS and OPC.

Previous preclinical cardiac arrest models in rats and pigs have revealed the decreased CI and CII function in the mitochondria of hippocampus (Yang et al., 2018; Lautz et al., 2019; Marquez et al., 2020). Although the global brain ischemia with reperfusion model was utilized, another study demonstrated CI suppression in mitochondria in the hippocampus and cortex during ischemia (Chomova et al., 2012; Borutaite et al., 2013). A previous study of an 8-min cardiac arrest mouse model with 60 min of reperfusion found decreased CI function but normal CII function in heart mitochondria, which suggests that CI is a more vulnerable site than CII in myocardium (Han et al., 2008). In contrast, a previous clinical study that investigated skeletal muscle biopsy in patients with out-of-hospital cardiac arrest identified significantly lower CI + II and ETS than in age-matched healthy controls, while CI showed no difference in muscle (Wiberg et al., 2020). The discrepancy in types of major complex dysfunction in mitochondria might originate from differences in modeling method and type of species, but the major difference appears to be the difference in target tissues. Mitochondrial diversity, including in the number, volume density, and gene expression profile, has been reported between tissues (Bugger et al., 2009; Kim et al., 2016). Additionally, several studies investigating the mitochondria have reported conflicting results between different tissues (heart vs. brain) for the same subject (Matsuura et al., 2017; Ji et al., 2021). As brain injury is the most devastating complication during PCAS that brings the main cause of mortality and long-term disability in survivors of cardiac arrest, investigation of mitochondrial dysfunction in cardiac arrest should be prioritized in brain (Sekhon et al., 2017; Perkins et al., 2021; Sandroni et al., 2021).

Surprisingly, at 3 h after ROSC, CI and CI + II respiration and ETS were rather increased in the CA and CA + VNS groups compared to the sham group. In addition, every increase in CI, CI + II, and ETS at 3 h was followed by a decrease at 6 h after ROSC. It can be assumed that mitochondrial dysfunction in PCAS represents delayed features. Consistent with our study, a previous study of global brain ischemia–reperfusion injury identified a slight increase in CI activity 1 h after reperfusion compared to controls (Chomova et al., 2012). Although the tissues were different, previous experimental studies on cardiac arrest have also identified that the activity of complex I-III was relatively unaffected and remained or rather than control levels until 30 min after ROSC (Han et al., 2008). In both global and focal brain ischemia models, the initial decline in mitochondrial respiration fully recovers during the first hour of reperfusion, but then delayed suppression of mitochondrial respiratory capacity is observed after 2–4 h of reperfusion, which supports our findings (Sims et al., 1998; Sims and Anderson, 2002). This deterioration might be attributed to the degradation or inactivation of the pyruvate dehydrogenase complex (Zaidan and Sims, 1993, 1997; Borutaite et al., 2013).

Several studies have demonstrated that VNS reduces mitochondrial dysfunction in ischemia–reperfusion injury by exerting antioxidant, anti-apoptotic, and anti-inflammatory effects (Jiang et al., 2015; Chunchai et al., 2016; Lai et al., 2019). While most studies have focused on mitochondrial dysfunction in myocardial ischemia–reperfusion injury, the present study focused on the brain, specifically the hippocampus, where most ischemia–reperfusion injury is generated during cardiac arrest with devastating sequelae in these patients (Sekhon et al., 2017). A possible mechanism of improvement in mitochondrial dysfunction in PCAS following VNS might be explained by the rapid recovery of cerebral blood flow. As demonstrated in our previous study, the no-reflow phenomenon, characterized by hypoperfusion following cerebral hyperemia, resolved with VNS treatment (Kim et al., 2019).

The current study, which implemented the VNS treatment immediately after ROSC for 3 h, highlights the technical feasibility of VNS in patients with ROSC after cardiac arrest. The approval of VNS by the United States of American Food and Drug Administration (FDA) as a treatment for patients with intractable focal seizures and its worldwide applications are some of its strengths (Johnson and Wilson, 2018; Yang and Phi, 2019; Yap et al., 2020).

This study has several limitations. First, the possible mechanisms of the restoration of mitochondrial dysfunction in the VNS group were not fully elucidated. Further detailed studies investigating the mechanisms of VNS on mitochondrial dysfunction, including the pyruvate dehydrogenase complex, are required. Second, mitochondrial dysfunction in the hippocampus was investigated, while neurological scores reflective of the cerebral cortex were evaluated. Cerebral injury in PCAS is a global injury in which the degree of injury in the various regions is similar and usually depends on ischemia time (Perkins et al., 2021; Sandroni et al., 2021). Due to the severity of our model, there was a limitation for the maze test, as motor function may unintentionally affect the outcome of spatial memory function. In addition, a previous study evaluated the mitochondrial dysfunction of similar CI activities in the hippocampus and cortex that assumes comparable injury in both sites (Chomova et al., 2012). Third, direct observation of nerve signal propagation or long-term stimulation effects, including the refractory period, were unidentifiable. However, the resulting decrease in heart rate in CA + VNS group was identified, which represents stimulation of the vagus nerve, and the VNS setting presented in this study (1-mA pulses of 10-ms duration at 1 Hz for 3 h) is usually an acceptable condition in both preclinical and clinical studies (Yap et al., 2020). Fourth, although body temperature was controlled with infrared heater to the extent possible, injection of drugs during the resuscitation period decreased the body temperature right after the ROSC period which might act as neuroprotective effect. Moreover, the brain temperature was unidentifiable due to the limitations of experimental setup. However, as both CA and CA + VNS group has been treated equally with identical protocol, we believe that the results of this study are still valid. Fifth, only male participants were included in our study. Although there are no differences in the prognosis of CA between men and women, further VNS studies in women might be needed.

## Conclusion

VNS improved mitochondrial dysfunction and neurological outcomes at 48 and 72 h during PCAS in a rat model of asphyxial cardiac arrest. Further study investigating the mechanisms of VNS on mitochondrial dysfunction in the brain, including the hippocampus, in cardiac arrest and the consequent neurological outcome must be conducted.

## Data Availability Statement

The raw data supporting the conclusions of this article will be made available by the authors, without undue reservation.

## Ethics Statement

The animal study was reviewed and approved by the Institutional Animal Care and Use Committee of the Seoul National University Bundang Hospital.

## Author Contributions

JL and YJ designed the study and prepared the protocol. SHK, IP, SeK, D-HJ, and JL carried out experiments. SHK, IP, and JL drafted and revised the manuscript. All authors participated in the interpretation of data, read, and approved the final manuscript.

## Conflict of Interest

The authors declare that the research was conducted in the absence of any commercial or financial relationships that could be construed as a potential conflict of interest.

## Publisher’s Note

All claims expressed in this article are solely those of the authors and do not necessarily represent those of their affiliated organizations, or those of the publisher, the editors and the reviewers. Any product that may be evaluated in this article, or claim that may be made by its manufacturer, is not guaranteed or endorsed by the publisher.
