# Supplementary material for: Vagus Nerve Stimulation Improves Mitochondrial Dysfunction in Post–cardiac Arrest Syndrome in the Asphyxial Cardiac Arrest Model in Rats
Source: Front Neurosci. 2022 May 26;16:762007. doi: 10.3389/fnins.2022.762007 (PMC9178208; doi:10.3389/fnins.2022.762007)
Supplement: Supplementary file 1 [file Data_Sheet_1.pdf]

Supplementary Data for

Vagus nerve stimulation improves mitochondrial dysfunction in post-cardiac arrest syndrome in the asphyxial cardiac arrest model in rats

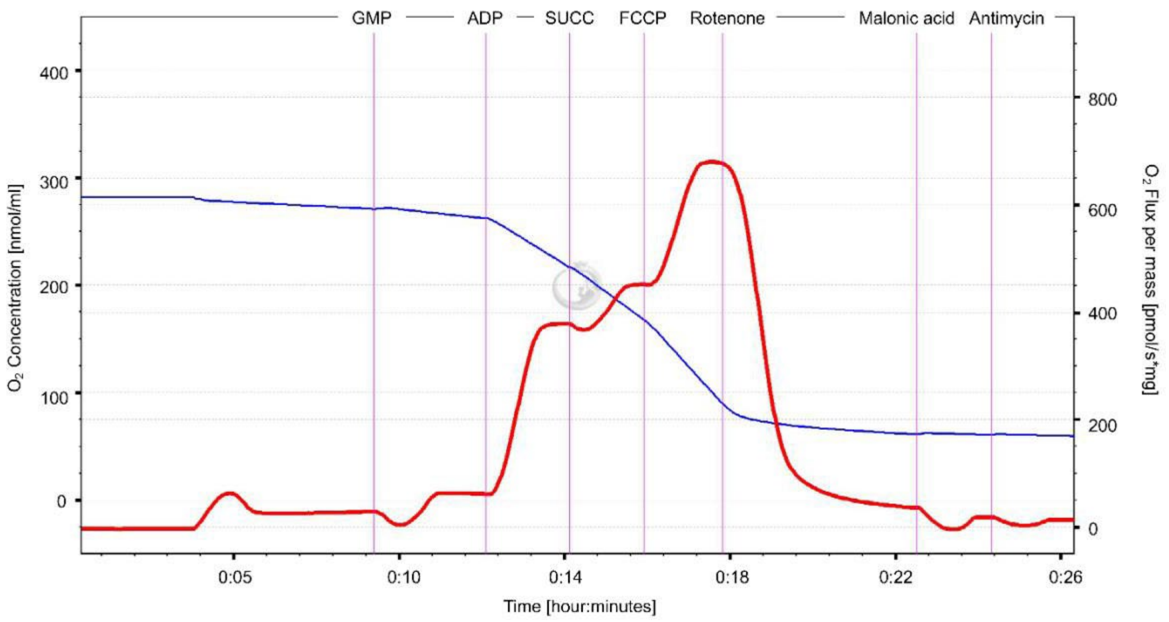

Supplementary Figure 1. Representative images of the SUIT protocol performed with high-resolution respirometry (OROBOROS). The blue line denotes the oxygen concentration (nmol/ml, left scale), and the red line indicates the oxygen flux in mass (pmol/s*mg, right scale). Reagents (upper scale) were added at the time (lower scale). GMP, glutamate, malate, pyruvate; ADP, adenosine diphosphate; SUCC, succinate; FCCP, carbonyl cyanide 4-(trifluoromethoxy) phenylhydrazone.

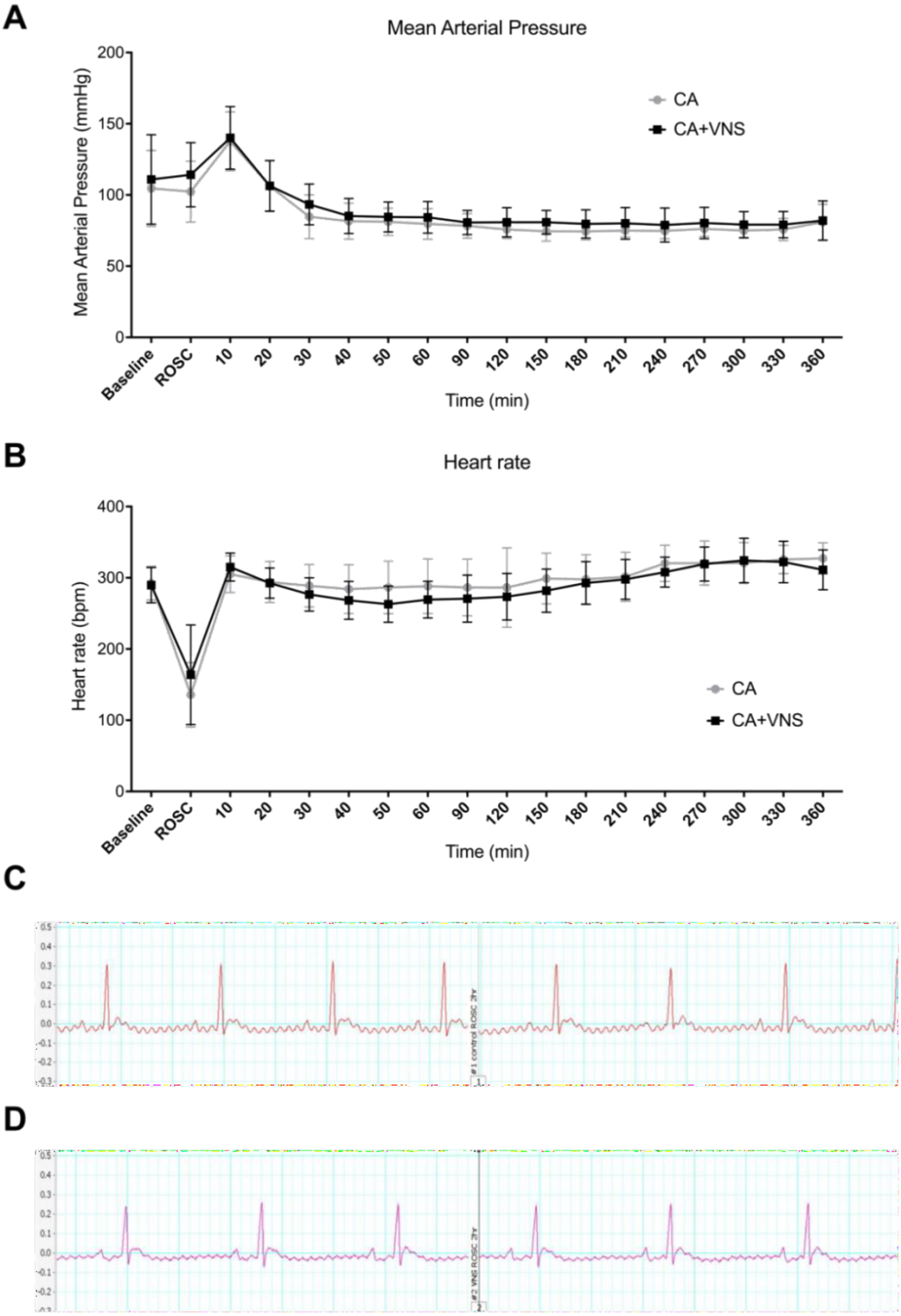

Supplementary Figure 2. Trends of hemodynamic variables from baseline to 6 hours after ROSC. (A) Trends of mean arterial pressure. $p < 0.0001$ between times, $p = 0.0701$ between groups, $p = 0.9914$ between times x groups; mixed-effects model. There was no significant difference in post hoc Bonferroni's multiple comparisons test. (B) Trends of heart rate. $p < 0.0001$ between times, $p = 0.6053$ between groups, $p = 0.0234$ between times x groups; mixed-effects model. There was no significant difference in post hoc Bonferroni's multiple comparisons test. Data are presented as the mean and standard deviation. Representative electrocardiography at 2 hours after the ROSC period in the (C) CA group and (D) CA+VNS group. One small square indicates 0.02 seconds, and the heart rate was recorded as 275 and 227 beats per minute, respectively.

Supplementary Table 1. Comparison of heart rate variables during experimental period.

Variables	CA (n=28)	CA+VNS (n=26)	*p value*
Baseline	290 [274-314]	285 [271-316]	0.7341
ROSC	128 [96-165]	134 [116-246]	0.3034
30 min	286 [261-314]	277 [258-292]	0.1886
60 min	287 [259-324]	267 [254-284]	0.1214
90 min	293 [259-325]	265 [245-293]	0.0783
120 min	306 [260-327]	267 [252-302]	0.0859
150 min	312 [266-350]	281 [264-308]	0.0961
180 min	303 [273-332]	297 [275-314]	0.6414

Variables are presented with median [IQR].
